# Symptomatic Subdural Hygroma Following Acinetobacter Meningitis in an Infant

**DOI:** 10.7759/cureus.48164

**Published:** 2023-11-02

**Authors:** Bárbara Pereira Neto, Rui Tuna, Luísa Sampaio, Ana Vilan

**Affiliations:** 1 Pediatrics, Centro Hospitalar de São João, Porto, PRT; 2 Neurosurgery, Centro Hospitalar de São João, Porto, PRT; 3 Neuroradiology, Centro Hospitalar de São João, Porto, PRT; 4 Neonatology, Centro Hospitalar de São João, Porto, PRT

**Keywords:** subdural collection, postmeningitis collection, neonatal seizures, meningitis, acinetobacter

## Abstract

We present a case of a full-term newborn with prenatal congenital heart disease, admitted to a level III neonatal intensive care unit. After undergoing a surgical palliation procedure, he experienced a complicated recovery, including nosocomial sepsis with isolation of *Acinetobacter nosocomialis* in both blood and cerebrospinal fluid. Subsequently, he developed focal clonic seizures that were refractory to antiepileptic drugs, and imaging studies revealed the presence of a subdural hygroma. Surgical drainage was performed, resulting in the resolution of the seizures. This report highlights the rare occurrence of *Acinetobacter* meningitis unrelated to neurosurgery and its progression to subdural hygroma in an infant, emphasizing the importance of recognizing such complications as potential causes of refractory seizures following infectious processes.

## Introduction

Subdural hygromas typically arise as a consequence of ventricular shunting; however, they can also manifest as a complication of bacterial meningitis [1,2]. In young infants, bacterial meningitis is predominantly caused by group B *Streptococcus* (GBS) and Gram-negative species [3]. While *Acinetobacter *meningitis has conventionally been linked to neurosurgical procedures [4], this case report delves into an unusual case of *Acinetobacter *meningitis unrelated to surgery, complicating with a subdural hygroma, shedding light on this atypical presentation.

## Case presentation

**Figure 1:**
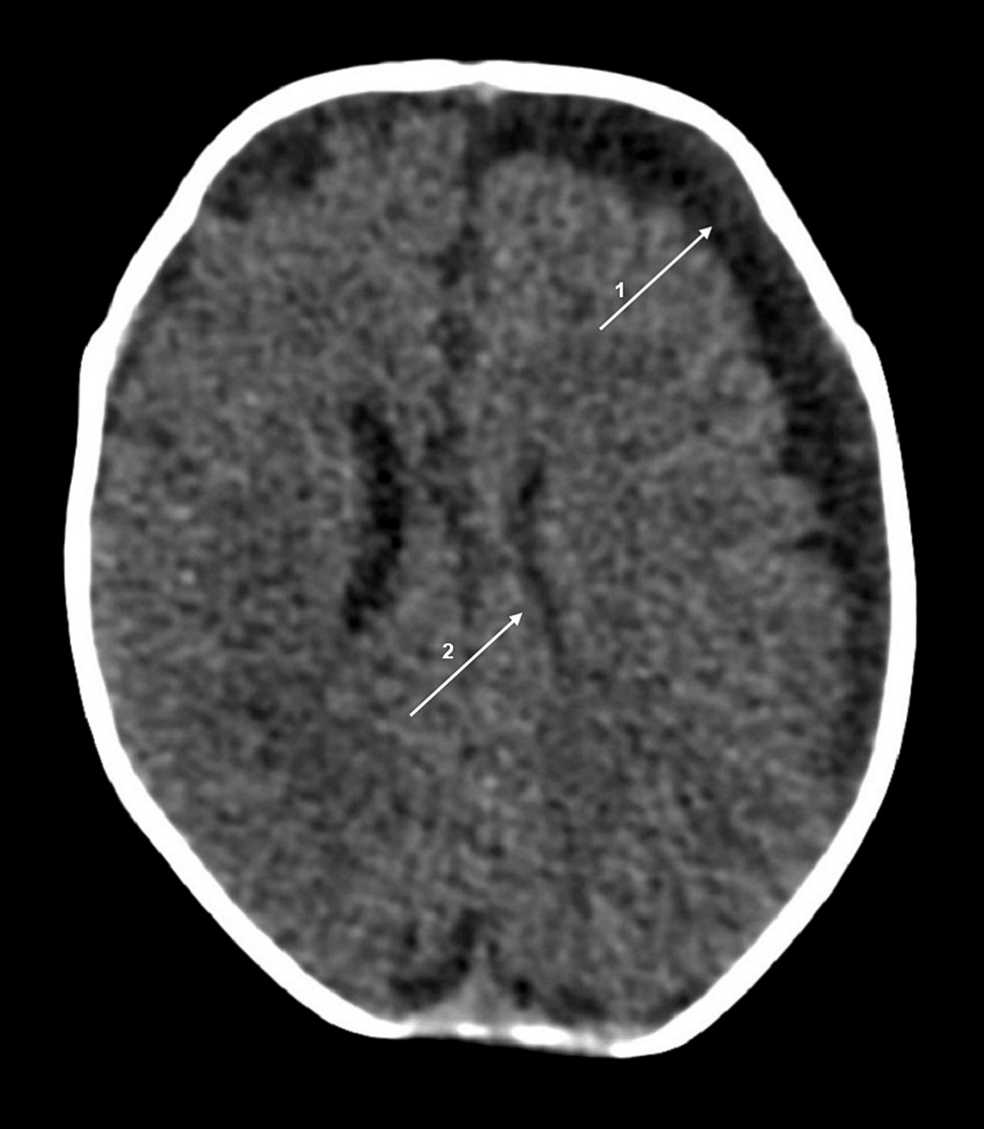
CT scan of the head Left frontotemporal hypodense effusion (arrow 1), with mass effect (sulci effacement and ventricular molding - arrow 2), compatible with hygroma.

A full-term newborn with prenatal undiagnosed hypoplastic left heart syndrome with partial anomalous pulmonary venous drainage was transferred to a level III neonatal intensive care unit soon after birth. He remained stable with prostaglandin E1 and a high-flow nasal cannula (maximum fraction of inspired oxygen = 0.25). A stage 1 procedure of surgical palliation was undertaken on the 35th day of life. He had a complicated recovery with failure of extubation, refractory ascites, and nosocomial sepsis with isolation of *Acinetobacter nosocomialis *in blood and cerebrospinal fluid (CSF) culture. Despite the appropriate antibiotherapy, the infant presented intermittent spikes of fever with negative septic screenings, including CSF. Three weeks later, focal clonic seizures of the right upper and lower limbs, with ocular movements and oxygen desaturation, were observed, with no response to phenobarbital and levetiracetam. Cranial ultrasound (cUS) revealed subdural collection on the left side, later confirmed on a CT scan of the head to be a hygroma (Figure 1).

As electroclinical seizures persisted, with repetitive ictal discharges from the left hemisphere and poor response to a continuous infusion of midazolam and lidocaine, surgical drainage was performed. Even though the fluid collection persisted on repeat cUS, seizures resolved. The overall status of the infant deteriorated with severe electrolytic imbalance due to his refractory ascites and actions were redirected to palliative care.

## Discussion

Subdural hygroma, also named “fluid collection” or “effusion” by various authors [1,5,6], is defined as an accumulation of CSF in the subdural space and may be secondary to ventricular shunting (the most common cause), brain injury, meningitis, or may be idiopathic, as a result of abnormal CSF absorption and rupture of the outer arachnoid membrane [1,2]. Postmeningitis subdural collection (a term that authors often wrongly use as a synonym of subdural empyema [7]) is a common complication of bacterial meningitis in infancy [8]. It occurs in 40% to 60% of infants [7] and is thought to be a consequence of the meningeal inflammatory reaction [8]. The most common agents of bacterial meningitis in infants are GBS and Gram-negative species, such as *Escherichia* *coli* [3]. *Acinetobacter *meningitis, on the other hand, occurs mainly following neurosurgery [4].

Subdural hygromas compress the underlying brain, which may be responsible for not only neurological symptoms but also developmental anomalies in pediatric age. Furthermore, this condition, opposing to other similar collections (such as benign peri-cerebral, subdural, extra-axial, or subarachnoid fluid collections of infancy), does not resolve spontaneously [5] and may develop into subdural hematomas (usually about one month after the appearance of the hygroma) [6]. The main symptoms are increased intracranial pressure, macrocrania, delayed development, hemiparesis, seizures, and decreased consciousness [1,6].

Imaging studies show an enlargement of the subdural space, which is filled with CSF [9]. Distinction from cerebral atrophy with cUS and CT alone is difficult but may be possible through contrast-enhanced MRI or perfusion single photon emission tomography (SPECT) [2].

The decision to adopt a conservative or surgical management is still controversial. Most subdural hygromas resolve spontaneously, thus the decision regarding the type of management is guided by clinical presentation and imaging findings [2], including the size of the lesion and its progression [5,6]. The most common indications for surgery are persistent or recurrent fever, seizures, and bulging fontanelle [7]. As such, quick diagnosis and drainage allow clinical resolution, as well as posterior normal brain development [5].

## Conclusions

The importance of this report lies in the rarity of both *Acinetobacter *meningitis unrelated to neurosurgery and its progression to subdural hygroma. As such, it emphasizes the need to be aware of these complications as a cause of seizures and their recognition in imaging studies, especially in the aftermath of an infectious process.
